# Plant Metabolites Drive Different Responses in Caterpillars of Two Closely Related *Helicoverpa* Species

**DOI:** 10.3389/fphys.2021.662978

**Published:** 2021-04-21

**Authors:** Longlong Sun, Wenhua Hou, Jiajia Zhang, Yuli Dang, Qiuyun Yang, Xincheng Zhao, Ying Ma, Qingbo Tang

**Affiliations:** ^1^The Institute of Chemical Ecology and College of Plant Protection, Henan Agricultural University, Zhengzhou, China; ^2^College of Agronomy, Henan Agricultural University, Zhengzhou, China

**Keywords:** *Helicoverpa armigera*, *Helicoverpa assulta*, plant primary metabolites, plant secondary metabolites, feeding preference, electrophysiological response, gustatory receptor neurons

## Abstract

The host acceptances of insects can be determined largely by detecting plant metabolites using insect taste. In the present study, we investigated the gustatory sensitivity and feeding behaviors of two closely related caterpillars, the generalist *Helicoverpa armigera* (Hübner) and the specialist *H. assulta* (Guenée), to different plant metabolites by using the single sensillum recording technique and the dual-choice assay, aiming to explore the contribution of plant metabolites to the difference of diet breadth between the two species. The results depicted that the feeding patterns of caterpillars for both plant primary and secondary metabolites were significantly different between the two *Helicoverpa* species. Fructose, glucose, and proline stimulated feedings of the specialist *H. assulta*, while glucose and proline had no significant effect on the generalist *H. armigera*. Gossypol and tomatine, the secondary metabolites of host plants of the generalist *H. armigera*, elicited appetitive feedings of this insect species but drove aversive feedings of *H. assulta*. Nicotine and capsaicin elicited appetitive feedings of *H. assulta*, but drove aversive feedings of *H. armigera*. For the response of gustatory receptor neurons (GRNs) in the maxillary styloconic sensilla of caterpillars, each of the investigated primary metabolites induced similar responding patterns between the two *Helicoverpa* species. However, four secondary metabolites elicited different responding patterns of GRNs in the two species, which is consistent with the difference of feeding preferences to these compounds. In summary, our results of caterpillars’ performance to the plant metabolites could reflect the difference of diet breadth between the two *Helicoverpa* species. To our knowledge, this is the first report showing that plant secondary metabolites could drive appetitive feedings in a generalist insect species, which gives new insights of underscoring the adaptation mechanism of herbivores to host plants.

## Introduction

The herbivorous insects use a variety of physiological mechanisms including pre-ingestive responses (i.e., chemosensory) (Bernays et al., 2000a; Glendinning, 2002), the post-ingestive response (Montandon et al., 1987; Behmer et al., 1999; Wright et al., 2010; Simões et al., 2012), and the detoxification processes (Mao et al., 2007; Tao et al., 2012; Bretschneider et al., 2016; Krempl et al., 2016; Tian et al., 2019) to cope with the plant metabolites, including primary and secondary metabolites. It is also accepted that herbivorous insects with different diet breadths have different capacities to discriminate these metabolites and extend to their decisions in host acceptance (Bernays et al., 2000b; Govind et al., 2010; Ahn et al., 2011; Liu et al., 2012; Kumar et al., 2014; Wang et al., 2017; Zhu et al., 2020). For example, the specialist herbivores were frequently reported to have more ability to metabolize or utilize the secondary metabolites than the generalists (Bernays et al., 2000b; Govind et al., 2010; Ahn et al., 2011; Liu et al., 2012; Kumar et al., 2014; Sun et al., 2019). Some specialists even detect the secondary metabolites as “token stimuli” for recognizing the specific host plant by using their chemoreceptors (Jermy, 1966; Schoonhoven, 1967; Renwick and Lopez, 1999; del Campo et al., 2001; Vickerman and de Boer, 2002; Miles et al., 2005). However, little attention has been paid in understanding whether the generalist herbivorous insects could recognize the plant metabolites from their hosts as “token‘ timuli.”

The dietary acceptance and host range of caterpillars might relate to the spectrum of the sensitivity of gustatory receptor neurons (GRNs) in the galeal styloconic sensilla to the plant metabolites (Jermy, 1966; Thompson, 1991; Bernays et al., 2000b; Wada-Katsumata et al., 2013; Sollai and Crnjar, 2019). Therefore, comparing feeding behaviors and taste responses between closely related species with different host ranges could contribute to understanding the host acceptability, diet breadth, and evolution of host adaptation (Sheck and Gould, 1996; Bernays et al., 2000b; Renwick, 2001; Liu et al., 2012; Sollai et al., 2014). The cotton bollworm *Helicoverpa armigera* (Hübner) (Lepidoptera: Noctuidae) and the tobacco budworm *Helicoverpa assulta* (Guenée) (Lepidoptera: Noctuidae) are two sympatric closely related herbivorous species. The former is an extreme generalist feeding on at least 161 host plant species in 49 plant families, including cotton, tomato, and tobacco (Zalucki et al., 1986; Fitt, 1989), whereas the latter is a specialist insect species feeding on the Solanaceae and several *Physalis* species, tobacco, and hot pepper on the natural field (Mitter et al., 1993). The two species could be hybridized to produce viable offspring under laboratory conditions (Wang and Dong, 2001) and are good models to investigate the interaction between plants and herbivorous insects (Tang et al., 2006, 2014; Ahn et al., 2011; Liu et al., 2012; Yang et al., 2017; Zhu et al., 2020).

In this study, we investigated the feeding preferences and the gustatory responses of caterpillars of the two *Helicoverpa* species to three plant primary metabolites, including fructose, glucose, and proline, and four plant secondary metabolites including gossypol, tomatine, nicotine, and capsaicin (Table 1). Fructose, glucose, and proline have been well known to be the energy source and phagostimulants for herbivorous insects (Albert et al., 1982; Bernays and Chapman, 2001; Liscia et al., 2004; Jiang et al., 2015; Mang et al., 2016). Gossypol and tomatine are plant secondary metabolites of cotton (Oliver et al., 1970; Montandon et al., 1987) and tomato, respectively (Barbour and Kennedy, 1991). Nicotine and capsaicin are plant secondary metabolites of tobacco and pepper, respectively (Pearson et al., 2019). Finally, we attempt to understand whether behavioral responses of two *Helicoverpa* species toward these plant metabolites corresponded with the diet breadth or not.

**TABLE 1:** The investigated plant metabolites and the corresponding host plants of the two Helicoverpa species.

**Species**	**Host plant**	**Secondary metabolites**
*H. armigera*	Cotton	Gossypol
	Tomato	Tomatine
	Tobacco	Nicotine
	Hot pepper	Capsaicin
*H. assulta*	Tobacco	Nicotine
	Hot pepper	Capsaicin

## Materials and Methods

### Insect Culture

All colonies of the *Helicoverpa* caterpillars were maintained in the laboratory at 75% ± 5% relative humidity and temperature (27 ± 1°C) under a controlled photoperiod (L16:D8). Both larvae of *H. armigera* and *H. assulta* were obtained from established laboratory colonies, which were reared on an artificial diet prepared from the following ingredients: wheat bran (150 g), soybean powder (80 g), yeast powder (25 g), casein (40 g), sorbic acid (3 g), ascorbic acid (3 g), sucrose (10 g), agar (20 g), vitamin composite powders (8 g), acetic acid (4 ml), and distilled water (1,500 ml) (Wu et al., 1990; Wu and Gong, 1997; Jiang et al., 2010). Adults were supplied with a 10% v/v solution of sucrose in water.

### Compounds

D-(-)-Fructose (Cas:57-48-7), D-(+)-glucose (Cas:50-99-7), L-proline (Cas:147-85-3), gossypol (Cas:303-45-7), capsaicin (Cas:2444-86-4), and tomatine (Cas:17406-45-0) were obtained from Beijing Solarbio Science & Technology Co., Ltd. Nicotine (Cas:54-11-5) was from Alfa Aesar. Ethanol absolute (Cas:64-17-5) and methanol (Cas:67-56-1) were from Tianjin De-En Chemical Reagent Co., Ltd. PVP (Cas:9003-39-8) was obtained from Tianjin Guangfu Fine Chemical Research Institute.

### Feeding Choice Assay

The dual-choice plant leaf disc bioassay was used to test the feeding preference of 5th instar larvae of the two *Helicoverpa* species as described by Wang et al. (2017). In general, leaf discs (10 mm diameter, about 156 mm^2^) were punched from fresh leaves of pepper *Capsicum frutescens* L., “Yu-Yi” (Solanaceae), which then were immersed in control or treatment solutions for 30 min. The plant primary metabolites D-fructose (1.0, 10, 30, 50 mM), D-glucose (1.0, 10, 30, 50 mM), and L-proline (0.1, 1.0, 10, 50 mM) were dissolved in water. The plant secondary metabolites gossypol, tomatine, and capsaicin were dissolved in solvent I (0.25% methanol, 5% ethanol, and 0.32% polyvinylpyrrolidone (PVP) in water) at 0.001, 0.01, 0.1, and 1.0 mM. Nicotine was dissolved in solvent II (0.16% PVP in water) at the concentrations of 0.001 mM, 0.01 mM, 0.1 mM, and 1.0 mM. The solvents were used as control.

Before the test, the fifth-instar caterpillars had been starved for about 8 h. A single caterpillar was placed in the center of a Petri dish (12 cm diameter) with a moist filter paper (Φ11 cm, Jiaojie^®^, China). Four solvent-treated leaf discs and four plant metabolite-treated leaf discs were arranged in an ABABABAB fashion around the dish. All Petri dishes were put under evenly distributed LED strip lights (8,000 Lm) at a temperature of 27 ± 1°C. Areas of all remnants of leaf discs were measured by using a transparency film (PP2910, 3M Corp.) when two of the four disks of either plant (A or B) had been consumed. Each caterpillar was tested only once. For the feeding preference assays, at least 90 replicates were conducted.

The feeding preference index was calculated as follows:

Preference index for control leaves (Pc) = area of control-disc consumed/(area of control-disc consumed + area of treatment-disc consumed)
Preference index for treatment leaves (Pt) = area of treatment-disc consumed/(area of control-disc consumed + area of treatment-disc consumed)

### Electrophysiological Recordings

The electrophysiological sensitivity of gustatory neurons in the styloconic sensilla on the maxillary galea of caterpillars to the plant metabolites was investigated using the single sensillum recording technique (van Loon, 1990; Roessingh et al., 1999). In brief, a head of an excised 5th instar caterpillar was mounted on a silver wire electrode which was connected to the input of a pre-amplifier (Syntech Taste Probe DTP-1, Hilversum, The Netherlands). The lateral or medial styloconic sensillum was recorded for the sensitivity to a stimulus at different concentrations. D-fructose, D-glucose, and L-proline were used as stimuli of primary metabolites with concentrations varying from 0.01, 0.1, 1.0 to 10 mM in 2 mM KCl. The previous work has shown that 2 mM KCl was an adequate electrolyte solvent for *Helicoverpa* caterpillars (Tang et al., 2015; Ma et al., 2016). The concentrations of gossypol, capsaicin, tomatine, and nicotine were from 0.001, 0.01, 0.1 to 1.0 mM. The first three stimuli were dissolved in solvent I, and nicotine was in solvent II. Both solvents for electrophysiological tests consist of 2 mM KCl. In case of synergistic interactions of mixed metabolites to GRNs, only a single sensillum in one caterpillar was tested for the responses to one kind of stimulus from low to high concentration. The electrolyte solvent was also tested as the control. For a single test, a glass microelectrode (tip diameter *ca.* 30 μm) filled with a stimulating solution was moved to contact with the tip of the lateral or medial sensillum with the aid of a micro-manipulator. The duration of a single stimulation was 2 s with a time interval of at least 3 min. Amplified signals were digitized by an A/D interface (IDAC-4, Syntech) and sampled into a personal computer. For each given concentration of a stimulus, the electrophysiological responses of at least 10 larvae were recorded.

The analysis of electrophysiological responses of styloconic sensilla to different stimuli was performed with the aid of AutoSpike v. 3.7 software (Syntech, Hilversum, The Netherlands). Briefly, in the case of the identification of GRNs, by measuring the amplitude, shape, and phasic temporal pattern, three impulse spikes were generally identified and labeled as small (S), intermediate (M), and large (L), which best responded to water, metabolites, and salt, respectively (Sollai et al., 2014; Ma et al., 2016). For distinguishing M-type spikes induced by primary metabolites and secondary metabolites, the intermediate 1 (M1) and intermediate 2 (M2) were assigned based on the spike amplitudes, correspondingly. The mean impulse frequency of each GRN in the first second (spk.s^–1^) was calculated.

### Statistical Analysis

For the comparison of feeding preferences of caterpillars between control and treatment, the value of the preference index was arcsine transformed and then subjected to the paired-sample *t*-test (*P* < 0.05).

All the values of the impulse frequency (spk.s^–1^) were square-root transformed before analysis. One-way ANOVA followed by the Student–Newman–Keuls (SNK) *post-hoc* test (*P* < 0.05) was used to compare the difference of the firing frequency of one type of GNR to one stimulus at different concentrations. The independent *t*-test was used to compare the mean impulse frequency of the same type of GRN between species. Finally, the GLM-Univariate was used to analyze the order of the mean impulse frequency of one type of GRNs to different compounds within species followed by the SNK *post-hoc* test for multiple comparisons (*P* < 0.05). All data were analyzed using SPSS software version 16.0.

## Results

### Electrophysiological Responses to Primary Metabolites

**FIGURE 1:**
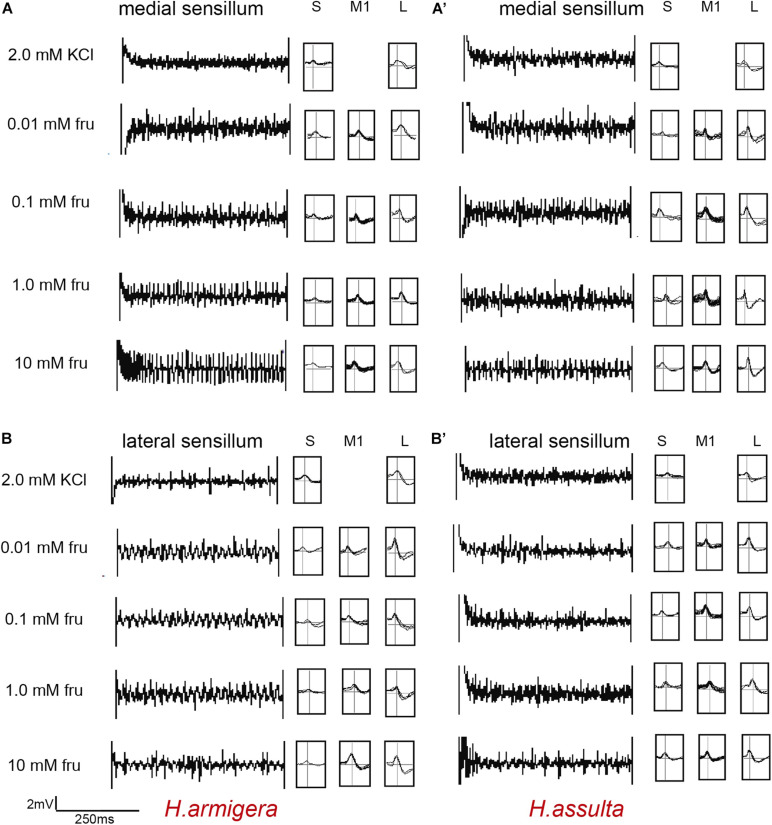
Representative traces and indentified GRNs from responses of the medial and lateral sensilla of *Helicoverpa* caterpillars to fructose. “S,” “M1,” and “L” in *H. armigera* **(A,A’)** and *H. assulta* **(B,B’)** represent the identified GRNs from recording traces based on the analysis of AutoSpike software. The time duration of each trace is 500 ms.

**FIGURE 2:**
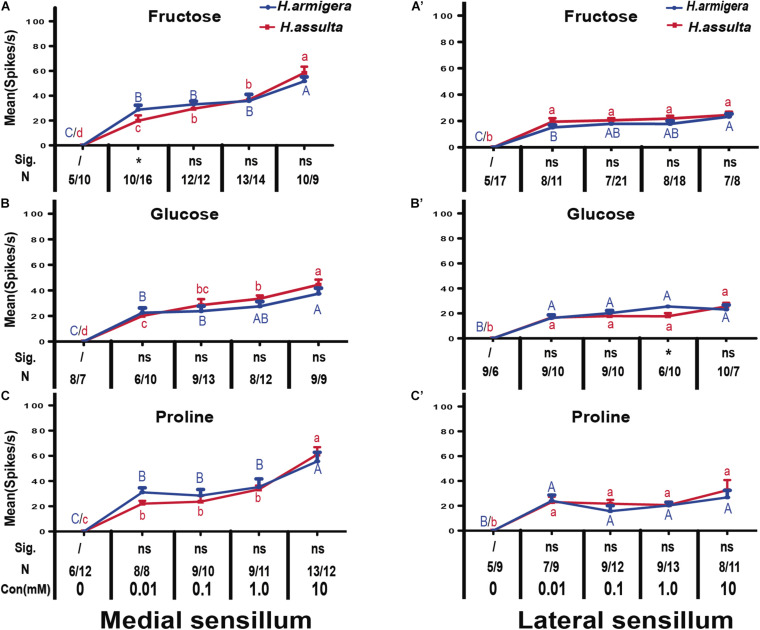
Comparisons of gustatory responses of “M1” GNRs in styloconic sensilla of *Helicoverpa* caterpillars to plant primary metabolites. Curves show the mean responding frequency ± SE of “M1” GNRs in the medial sensillum **(A–C)** and in the lateral sensillum **(A’–C’)** of *Helicoverpa* caterpillars to plant primary metabolites from 0.01 to 10 mM. Different capital letters and lowercase letters represent the mean responding frequencies of “M1” GNRs were significantly different in response to one primary metabolite at different concentrations in caterpillars of *H. armigera* and *H. assulta*, respectively (*post-hoc* SNK test of ANOVA: *P* < 0.05). Independent *t*-test was used to compare the difference of the mean responding frequency of “M1” GNRs to the same compound at the same concentration between the two *Helicoverpa* species. “Sig.” represents the levels of difference. “ns”: no significant different (*P* > 0.05); “*” represents the difference was significant at the 0.05 level. “N” represents the number of tested caterpillars of *H. armigera*/*H. assulta*.

In most recordings, three types of GRNs were identified from both medial and lateral sensilla of two *Helicoverpa* species in response to three plant primary metabolites, labeled as the “S” GRNs, “M1” GRNs, and “L” GRNs which best responded to water, primary metabolites, and salt, respectively (e.g., see identified representative GRNs in Figure 1). In the medial sensillum, the responses of “M1” GRNs of *H. armigera* caterpillars to each primary metabolite increased with the concentration increasing from 0, 0.01 mM, 0.1 mM, 1.0 mM to 10 mM [Figure 2A, one-way ANOVA of fructose: *F*_(__4, 45)_ = 37.393, *P* < 0.0001; Figure 2B, glucose: *F*_(__4, 35)_ = 51.272, *P* < 0.0001; Figure 2C, proline: *F*_(__4, 40)_ = 29.965, *P* < 0.0001]. The mean response frequencies of “M1” GRNs of *H. armigera* induced by 10 mM fructose, 10 mM glucose, and 10 mM proline were 51.70 ± 3.490 spk.s^–1^, 37.44 ± 4.378 spk.s^–1^, and 55.62 ± 7.161 spk.s^–1^, respectively.

Similarly, “M1” GRNs in the medial sensillum of *H. assulta* also showed increasing responses to each primary metabolite with increasing concentrations [*H. assulta* in Figure 2A’; one-way ANOVA of fructose: *F*_(__4, 56)_ = 99567, *P* < 0.0001; Figure 2B’, glucose: *F*_(__4, 46)_ = 55.164, *P* < 0.0001; Figure 2C’, proline: *F*_(__4, 48)_ = 93.889, *P* < 0.0001]. The mean response frequency of “M1” GRNs in the medial sensillum of *H. assulta* to 10 mM fructose, 10 mM glucose, and 10 mM proline were 58.44 ± 5.430 spk.s^–1^, 44.44 ± 4.045 spk.s^–1^, and 61.0 ± 6.881 spk.s^–1^, respectively. The responses of “M1” GRNs in the medial sensillum to one stimulus with the same concentration were always not significantly different between the two *Helicoverpa* species (Figures 2A–C, all comparisons: *P* > 0.05) except fructose at 0.01 mM which induced a significantly higher response of “M1” GRNs in *H. armigera* than that in *H. assulta* (Figure 2A, independent-sample *t*-test: df = 24, *t* = 2.411, *P* = 0.024). In the lateral sensillum, in contrast, the responses of “M1” GRNs to the three primary metabolites were low and the responses were similar between caterpillars of the two species (Figures 2A’–C’).

We also compared the general responding patterns of “M1” GRNs in one sensillum within the same species to the three primary metabolites using the GLM-Univariate with compounds and concentration as the fixed factors. It shows that the responses of “M1” GRNs in the medial sensillum of *H. armigera* caterpillars to the three compounds were significantly affected by both compounds and concentration (GLM-Univariate: compounds, df = 2, *F* = 4.199, *P* = 0.017; concentration, df = 4, *F* = 107.877, *P* < 0.0001). Analysis of the SNK *post-hoc* test showed that the responses of “M1” GRNs in the medial sensillum of *H. armigera* to glucose were significantly lower than those to fructose and proline (SNK *post-hoc* test: *P* < 0.05). However, for *H. assulta* caterpillars, the responses of “M1” GRNs in medial sensillum of *H. assulta* to the three compounds were not significantly affected by compound (GLM-Univariate: compounds, df = 2, *F* = 1.040, *P* = 0.356; concentration, df = 4, *F* = 234.979, *P* < 0.0001). Similarly, the responses of “M1” GRNs in lateral sensillum in both *Helicoverpa* species to the three compounds were also not significantly affected by compounds but affected significantly by concentrations (GLM-Univariate of *H. armigera*: compounds, df = 2, *F* = 0.563, *P* = 0.571; concentration, df = 4, *F* = 88.709, *P* < 0.0001; GLM-Univariate of *H. assulta*: compounds, df = 2, *F* = 1.630, *P* = 0.199; concentration, df = 4, *F* = 22.90, *P* < 0.0001).

**FIGURE 3:**
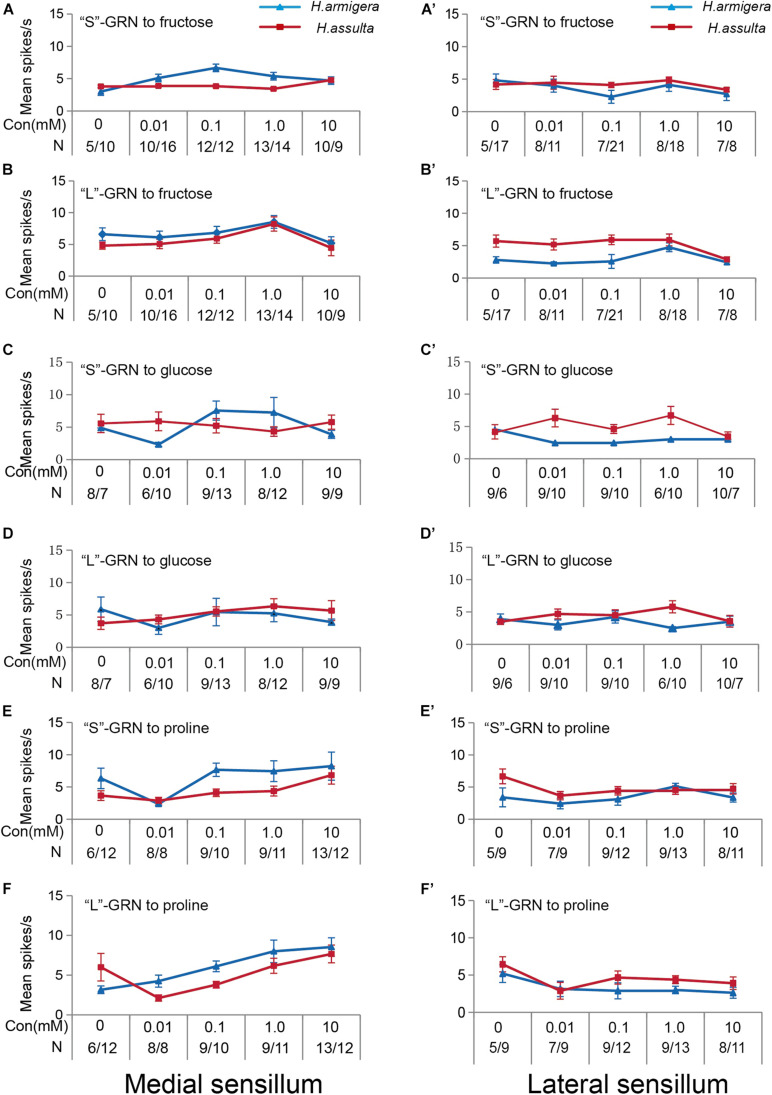
Responses of “S” and “L” GRNs in the styloconic sensilla of *Helicoverpa* caterpillars to the three primary metabolites. **(A,C,E)**: responses of the indentified “S” GRNs from the medial sensillum to fructose, glucose, and proline, respectively; **(B,D,F)**: responses of the indentified “L” GRNs from the medial sensillum to fructose, glucose, and proline, respectively; **(A’,C’,E’)**: responses of the indentified “S” GRNs from the lateral sensillum to fructose, glucose, and proline, respectively; **(B’,D’,F’)**: responses of the indentified “L” GRNs in the lateral sensillum to fructose, glucose, and proline, respectively.

The three primary metabolites also induced responses of “S” GRNs and “L” GRNs in both sensilla of the two *Helicoverpa* species. While the responses of the two types of GRNs to each compound were low with a non-significant change among different concentrations (SNK test after ANOVA for each compound: *P* > 0.05) (Figure 3).

### Feeding Preferences for Primary Metabolites

**FIGURE 4:**
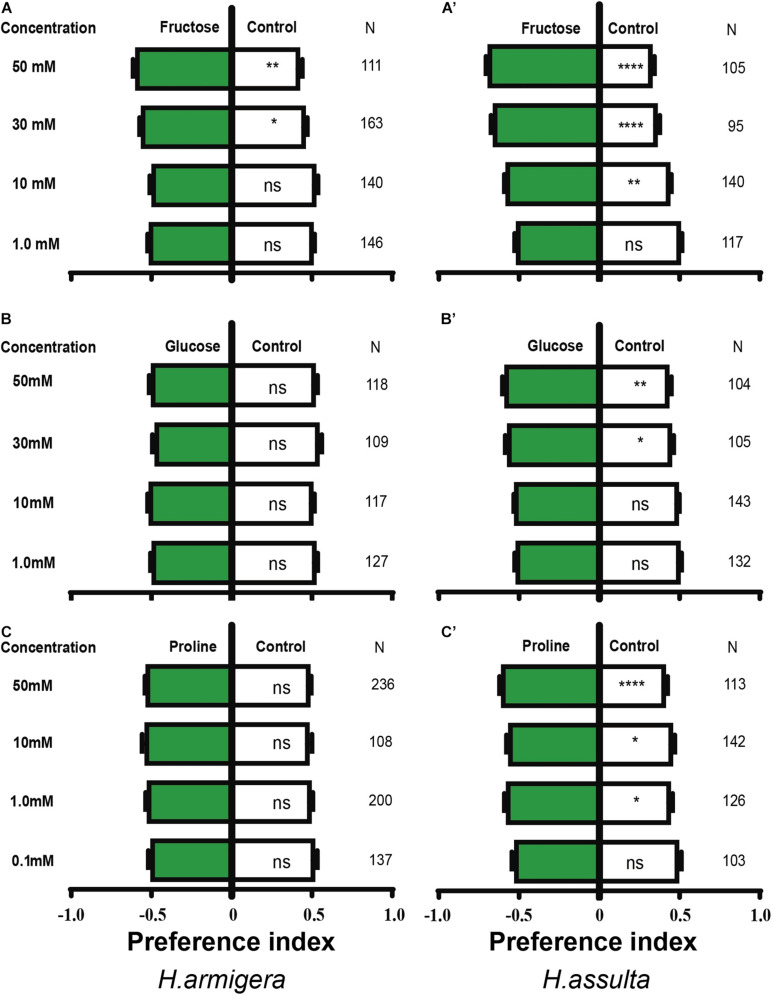
Effects of three primary metabolites on the feeding preferences of *Helicoverpa* caterpillars. The preference indexes of caterpillars for primary metabolites (green bars) and for control leaves (white bar) were compared. The dual-choice assay was used to test the feeding preference for control and pepper leaves treated by primary metabolites and water, respectively. **(A–C)**: *H. armigera* caterpillars; **(A’–C’)**: *H. assulta* caterpillars. A paired-sample *t*-test was used to compare the means of the preference indices between treatment and control. “*,” “**,” and “****” represent that the difference was significant at 0.05, 0.01, and 0.0001 levels, respectively. NS: non-significant difference. N: the number of tested caterpillars.

The high concentration of fructose drove obvious appetitive feedings of both *H. armigera* caterpillars [Figure 4A; paired-sample *t*-test: 30 mM, *t*(162) = −1.999, *P* = 0.047; 50 mM, *t*(110) = −2.88, *P* = 0.005] and *H. assulta* caterpillars [Figure 4A’; paired-sample *t*-test: 10 mM, *t*(139) = −3.329, *P* = 0.002; 30 mM, *t*(94) = −5.704, *P* < 0.0001; 50 mM, *t*(104) = −7.116, *P* < 0.0001]. However, glucose showed no obvious effect on the feeding of *H. armigera* at the given concentrations [Figure 4B; paired-sample *t*-test: 1 mM, *t*(126) = 0.700, *P* = 0.485; 10 mM, *t*(116) = −0.218, *P* = 0.828; 30 mM, *t*(108) = 1.358, *P* = 0.177; 50 mM, *t*(117) = 0.522, *P* = 0.602], while it drove appetitive feedings of *H. assulta* caterpillars at high concentrations [Figure 4B’; paired-sample *t*-test: 30 mM, *t*(104) = −2.308, *P* = 0.023; 50 mM, *t*(103) = −2.865, *P* = 0.004].

Similarly, proline had no significant effect on the feeding of *H. armigera* caterpillars [Figure 4C; paired-sample *t*-test: 0.1 mM, *t*(136) = 0.400, *P* = 0.690; 1.0 mM, *t*(199) = −0.803, *P* = 0.423; 10 mM, *t*(107) = −1.020, *P* = 0.310; 50 mM, *t*(235) = −1.223, *P* = 0.223], while feeding preferences of *H. assulta* were significantly elicited at 1.0, 10, and 50 mM [Figure 4C’; paired-sample *t*-test: 1.0 mM, *t*(125) = −2.541, *P* = 0.012; 10 mM, *t*(141) = −2.252, *P* = 0.026; 50 mM, *t*(112) = −4.276, *P* < 0.0001].

### Electrophysiological Responses to Secondary Metabolites

**FIGURE 5:**
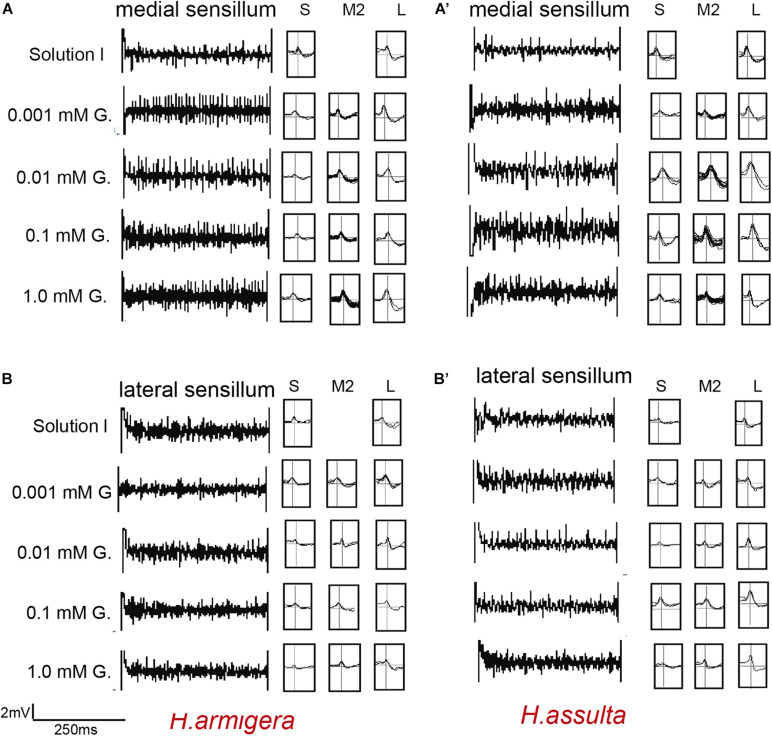
Representative traces and indentified GRNs from the responses of the styloconic sensilla of *Helicoverpa* caterpillars to gossypol. “S,” “M2,” and “L” GRNs in *H. armigera* **(A,B)** and *H. assulta* **(A’,B’)** represent the identified GRNs from recording traces based on the analysis of AutoSpike software. G: gossypol. The time duration of each trace is 500 ms.

**FIGURE 6:**
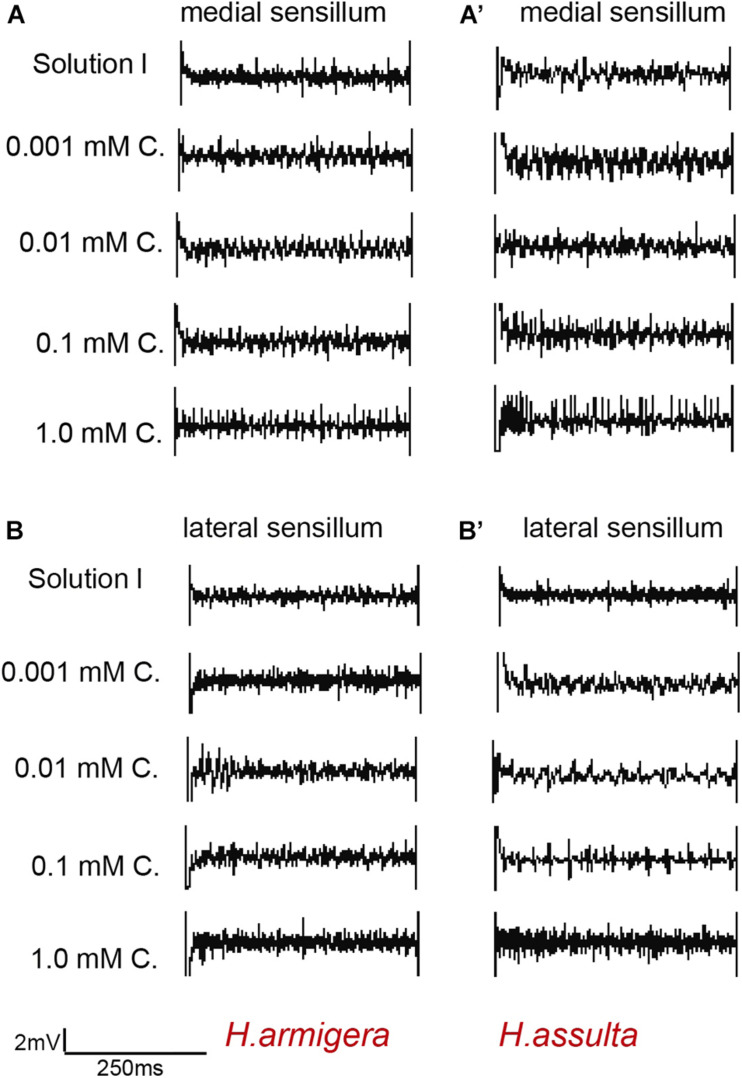
Representative traces from responses of the styloconic sensillum of *Helicoverpa* caterpillars to capsaicin. **(A,A’)**: medial sensillum of *H. armigera* and *H. assulta* to capsaicin, respectively; **(B,B’)**: lateral sensillum of *H. armigera* and *H. assulta* to capsaicin, respectively. C: capsaicin. The time duration of each trace is 500 ms.

The four investigated plant secondary metabolites induced high responses of the medial sensillum (e.g., see representative traces in Figures 5A,A’, 6A,A’) compared to the relatively low responses of the lateral sensillum of the two *Helicoverpa* species (e.g., see traces in Figures 5B,B’, 6B,B’). Three types of GRNs, in most traces, were identified in the responses of both sensilla to the four compounds, including the “S” GRNs, the “M2” GRNs, and the “L” GRNs, which best responded to water, the secondary metabolites, and salt, respectively (e.g., see representative identified GRNs in Figure 5).

**FIGURE 7:**
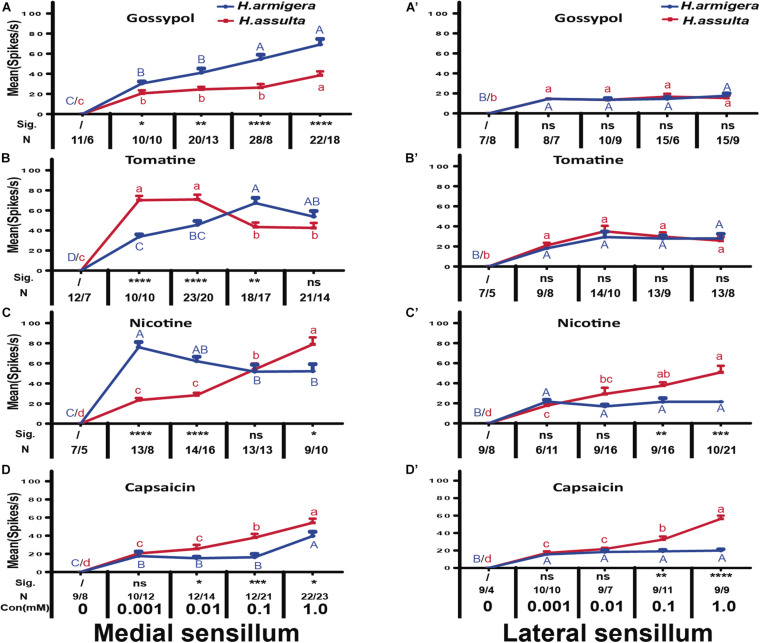
Comparisons of gustatory responses of “M2” GNRs in styloconic sensilla of *Helicoverpa* caterpillars to different plant secondary metabolites. Curves show the mean responding frequency ± SE of “M2” GNRs in the medial sensillum **(A–D)** and in the lateral sensillum **(A’–D’)** of *H. armigera* and *H. assulta* caterpillars to secondary metabolites from 0.001 to 1.0 mM. Different capital letters and lowercase letters represent the mean responding frequencies of “M2” GNRs which were significantly different in response to one compound at different concentrations in caterpillars of *H. armigera* and *H. assulta*, respectively (*post-hoc* SNK test of ANOVA: *P* < 0.05). Independent *t*-test was used to compare the difference of the mean responding frequency of “M2” GNRs to the same compound at the same concentration between the two Helicoverpa species. “Sig.” represents the levels of difference. “ns”: no significant different (*P* > 0.05); “*,” “**,” “***,” and “****” represent that the difference was significant at the 0.05, 0.01, 0.001, and 0.0001 levels, respectively. “N” represents the number of tested caterpillars of *H. armigera*/ *H. assulta*.

In general, the responses of “M2” GRNs in both sensilla of the two *Helicoverpa* species induced by four secondary metabolites were high, while the responses of “S” GRNs and “L” GRNs in both sensilla induced by four secondary metabolites were relatively low. The responses of “M2” GRNs in the medial sensillum to each of the four plant secondary metabolites were different between the two species. Gossypol induced higher levels of response of “M2” GRNs in medial sensillum of *H. armigera* caterpillars than that of *H. assulta* (Figure 7A, independent-sample *t*-test of 0.001 mM: df = 18, *t* = 2.79, *P* = 0.0121; 0.01 mM: df = 31, *t* = 3.19, *P* = 0.0033; 0.1 mM: df = 34, *t* = 3.70, *P* = 0.0001; 1.0 mM: df = 38, *t* = 4.45, *P* = 0.0001). Tomatine at 0.001 mM and 0.01 mM induced lower responses of “M2” GRNs in *H. armigera* than those in *H. assulta* caterpillars (Figure 7B, independent-sample *t*-test of 0.001 mM: df = 18, *t* = −7.65, *P* < 0.0001; 0.01 mM: df = 41, *t* = −4.06, *P* = 0.0002) but elicited higher levels of response at high concentration in *H. armigera* than that of *H. assulta* (Figure 7B, independent-sample *t*-test of 0.1 mM: df = 33, *t* = 3.36, *P* = 0.002; 1.0 mM: df = 33, *t* = 1.26, *P* = 0.2128).

Different from tomatine, nicotine at 0.001 mM and 0.01 mM induced higher levels of responses of “M2” GRNs in the medial sensillum of *H. armigera* than those of *H. assulta* (Figure 7C, independent-sample *t*-test of 0.001 mM: df = 19, *t* = 8.69, *P* < 0.0001; 0.01 mM: df = 28, *t* = 6.91, *P* < 0.0001) but elicited lower levels of response at 1.0 mM in *H. armigera* than that of *H. assulta* caterpillars(Figure 7C, 1.0 mM: df = 17, *t* = −2.88, *P* = 0.0105). Capsaicin elicited relatively lower levels of responses of “M2” GRNs in the medial sensillum of *H. armigera* caterpillars than those of *H. assulta* caterpillars (Figure 7D, independent-sample *t*-test of 0.01 mM: df = 20, *t* = −2.35, *P* = 0.0271; 0.1 mM: df = 31, *t* = −4.05, *P* = 0.0003; 1.0 mM: df = 43, *t* = −2.33, *P* = 0.0245).

For responses of “M2” GRNs in the lateral sensillum, it showed that gossypol and tomatine induced low and similar responses of “M2” GRNs between the two *Helicoverpa* species (Figure 7A’ 0.001 mM gossypol: df = 13, *t* = 0.01, *P* = 0.99; 0.01 mM gossypol: df = 17, *t* = −0.02, *P* = 0.98; 0.1 mM gossypol: df = 19, *t* = −0.70, *P* = 0.49; 1.0 mM gossypol: df = 22, *t* = 0.54, *P* = 0.60; Figure 7B’, 0.001 mM tomatine: df = 15, *t* = −0.92, *P* = 0.37; 0.01 mM tomatine: df = 22, *t* = −0.92, *P* = 0.37; 0.1 mM tomatine: df = 20, *t* = −0.40, *P* = 0.69; 1.0 mM tomatine: df = 19, *t* = 0.18, *P* = 0.86). However, the responses of “M2” GRNs in the lateral sensillum to both nicotine and capsaicin at 0.1 mM and 1.0 mM were higher in *H. assulta* caterpillars than those of *H. armigera* (Figure 7C’, 0.1 mM nicotine: df = 23, *t* = −3.30, *P* = 0.0031; 1.0 mM nicotine: df = 29, *t* = −4.13, *P* = 0.0004; Figure 7D’, 0.1 mM capsaicin: df = 18, *t* = −3.23, *P* = 0.0047; 1.0 mM capsaicin: df = 16, *t* = −9.27, *P* < 0.0001).

**FIGURE 8:**
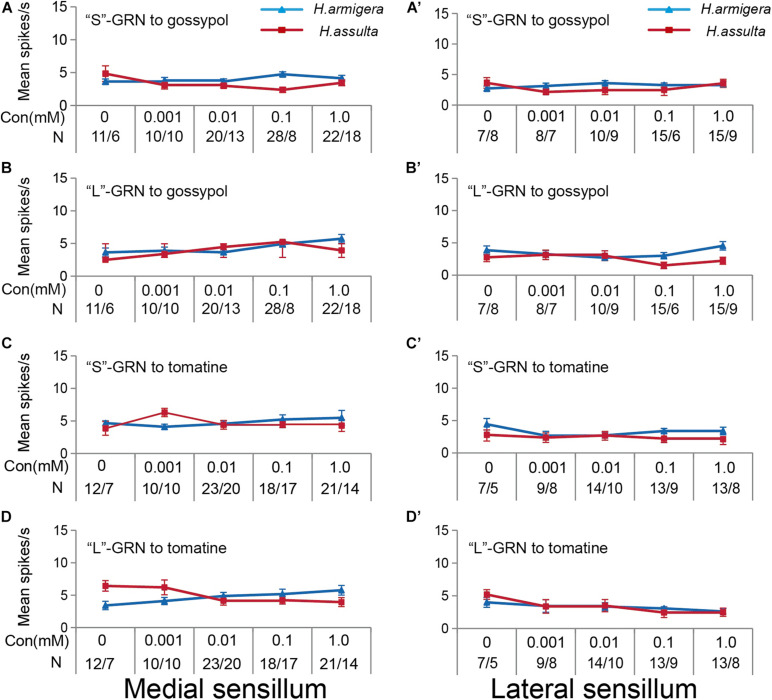
Responses of “S” and “L” GRNs in the styloconic sensilla of *Helicoverpa* caterpillars to gossypol and tomatine. **(A,A’)**: responses to gossypol of the indentified “S” GRNs from the medial and the lateral sensillum, respectively; **(B,B’)**: responses to gossypol of the indentified “L” GRNs from the medial and the lateral sensillum, respectively; **(C,C’)**: responses to tomatine of the indentified “S” GRNs from the medial and the lateral sensillum, respectively; **(D,D’)**: responses to tomatine of the indentified “L” GRNs from the medial and the lateral sensillum, respectively.

**FIGURE 9:**
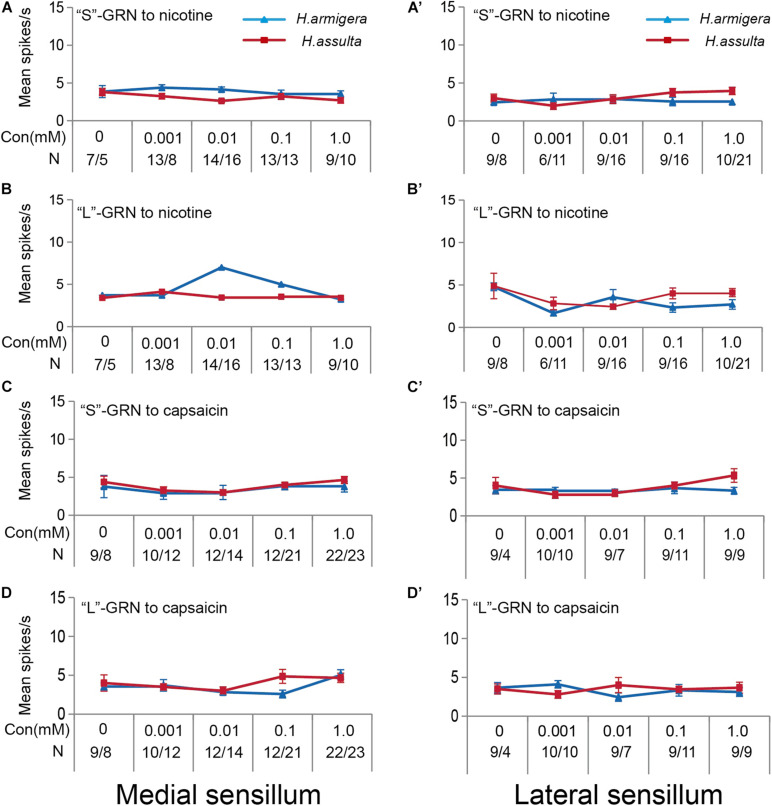
Responses of “S” and “L” GRNs in the styloconic sensilla of Helicoverpa caterpillars to nicotine and capsaicin. **(A,A’)**: responses to nicotine of the indentified “S” GRNs from the medial and lateral sensillum, respectively; **(B,B’)**: responses to nicotine of the indentified “L” GRNs from the medial and lateral sensillum, respectively; **(C,C’)**: responses to capsaicin of the indentified “S” GRNs from the medial and the lateral sensillum, respectively; **(D,D’)**: responses to capsaicin of the indentified “L” GRNs from the medial and the lateral sensillum, respectively.

Four plant secondary metabolites also induced responses of “S” GRNs and “L” GRNs in both sensilla of the two *Helicoverpa* species. While the responses of the two GRNs to each compound were low with non-significant change among different concentrations (SNK test after ANOVA for each compound: *P* > 0.05) (gossypol: Figures 8A,A’, B,B’; tomatine: Figures 8C,C’, D,D’; nicotine: Figures 9A,A’, B,B’; capsaicin: Figures 9C,C’, D,D’).

By comparing the responses of “M2” GRNs within one sensillum to the four secondary metabolites, it shows that the responses were significantly affected by both compounds and concentrations in either *Helicoverpa* species (GLM-univariate analysis of medial sensillum of *H. armigera*: compounds, df = 3, *F* = 39.814, *P* < 0.0001; concentrations, df = 4, *F* = 188.576, *P* < 0.0001; compounds × concentrations, df = 12, *F* = 7.659, *P* < 0.0001; medial sensillum of *H. assulta*: compounds, df = 3, *F* = 19.4448, *P* < 0.0001; concentrations, df = 4, *F* = 151.172, *P* < 0.0001; compounds × concentrations, df = 12, *F* = 17.406, *P* < 0.0001) (Table 2). However, the ranks of the general responding frequency between the two species were different. The response of “M2” GRNs in medial sensillum of *H. armigera* was the strongest to nicotine, followed by gossypol and tomatine, then low response to capsaicin (Table 3). However, for *H. assulta*, tomatine induced the strongest response of “M2” GRNs in the medial sensillum, followed by nicotine, capsaicin, and gossypol (Table 3). For the “M2” GRNs in the lateral sensillum between the two species, tomatine induced relatively stronger responses than those induced by gossypol, nicotine, and capsaicin in *H. armigera*, whereas gossypol induced the lowest responses compared to those by other three compounds in *H. assulta* (Table 3).

**TABLE 2:** Analysis of variance of the gustatory responses of “M2” GRNs in styloconic sensilla of *Helicoverpa* spp. partitioning effects of compounds and concentrations (GLM-Univariate analysis).

**Source of variation**		**Medial sensillum**			**Lateral sensillum**			
	**df**	**MS**	**F**	**Sig.**	**df**	**MS**	**F**	**Sig.**
**(A) *H. armigera***								
Com.	3	73.636	39.814	<0.0001	3	7.276	9.424	<0.0001
Con.	4	348.751	188.576	<0.0001	4	126.464	163.801	<0.0001
Com. × Con.	12	14.166	7.659	<0.0001	12	0.962	1.245	0.255
Error	276	1.849			180	0.772		
**(B) *H. assulta***								
Com.	3	31.01	19.4448	<0.0001	3	16.636	11.027	<0.0001
Con.	4	241.048	151.172	<0.0001	4	138.273	91.657	<0.0001
Com. × Con.	12	17.406	10.916	<0.0001	12	5.381	3.567	<0.0001
Error	235	1.595			172	1.509		

**Raw data of response frequencies were square-root transformed before analysis to meet the assumptions of GLM. Com.: compounds including gossypol, tomatine, nicotine, and capsaicin. Con.: concentrations.**

**TABLE 3:** Multiple comparisons of gustatory responses of “M2” GRNs in styloconic sensilla of *Helicoverpa* spp. to different plant secondary metabolites.

**Compounds**	**Medial sensillum**		**Lateral sensillum**	
	***H. armigera***	***H. assulta***	***H. armigera***	***H. assulta***
Gossypol	45.90 ± 29.09 b	28.07 ± 19.98 d	13.35 ± 8.36 b	11.87 ± 7.92 b
Tomatine	44.36 ± 28.21 b	50.81 ± 27.85 a	23.20 ± 16.76 a	24.83 ± 15.59 a
Nicotine	53.55 ± 29.18 a	40.94 ± 28.49 b	16.05 ± 10.42 b	32.44 ± 26.03 a
Capsaicin	21.92 ± 20.52 c	33.95 ± 23.84 c	14.57 ± 8.67 b	28.98 ± 19.28 a

**Data are shown as general mean responding frequency ± SE (spk.s^–1^) of “M2” GRNs to stimulus. Raw data of responding frequencies were square-root transformed before analysis. The SNK post-hoc test was used to the difference of response of “M2” GRNs in the same sensillum to different compounds (P < 0.05).* Different lowcase letters in a vertical column represent the difference is significant (P < 0.05).*

### Feeding Preferences for Plant Secondary Metabolites

**FIGURE 10:**
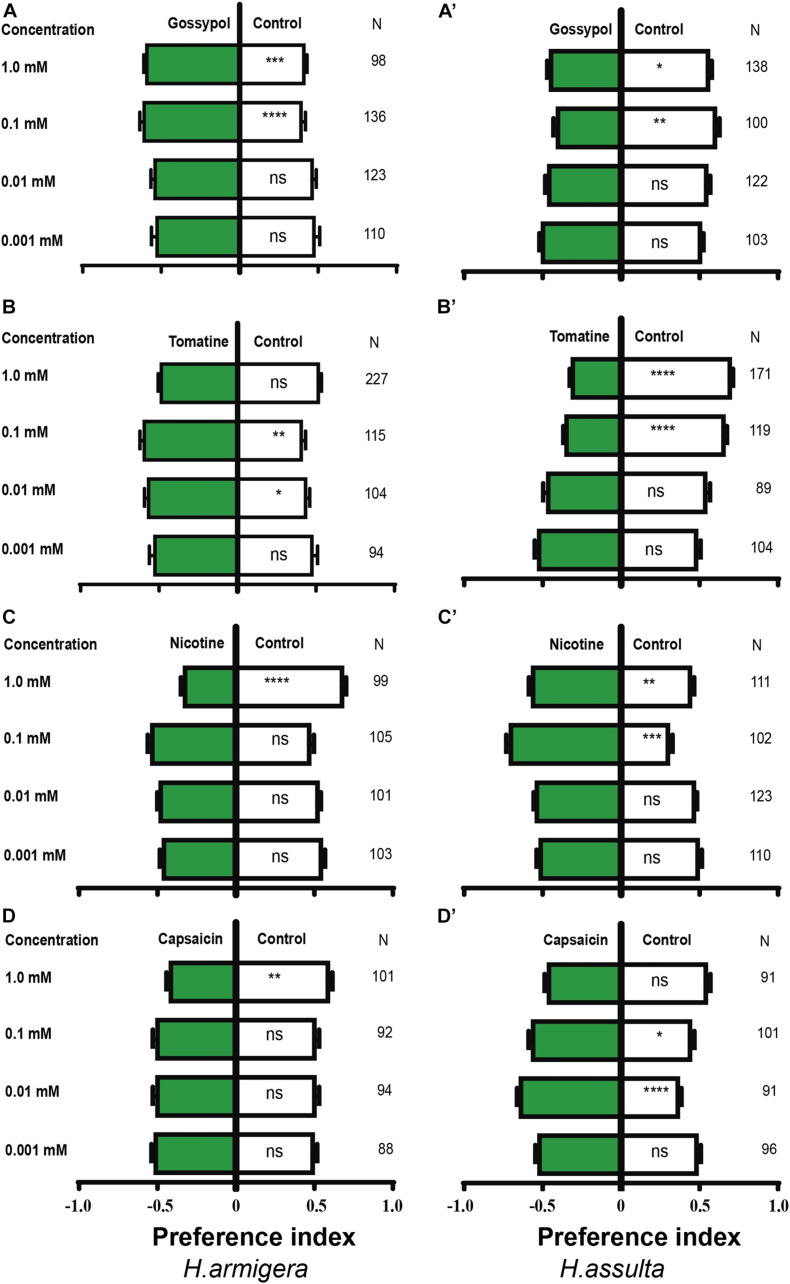
Effects of secondary metabolites on the feeding preferences of *Helicoverpa* caterpillars. The preference indexes of caterpillars for control leaves (white bar) and for secondary metabolites treated leaves (green bars) were compared by using the paired-sample *t*-test. The dual-choice assay was used to test the feeding preference for pepper leaves treated by secondary metabolites at different concentrations and for control (electrolyte-treated) leaves. Electrolyte in **(A,A’,B,B’,C,C’)** was solvent I (0.25% methanol, 5% ethanol, and 0.32% PVP in water). Electrolyte in **(D,D’)** was solvent II (0.16% PVP in water). **(A–D)**: *H. armigera* caterpillars; **(A’–D’)**: *H. assulta* caterpillars. “*,” “**,” “***,” and “****” represent that the difference was significant at the 0.05, 0.01, 0.001, and 0.0001 level, respectively. ns: non-significant difference. N: the number of tested caterpillars.

Gossypol at 0.1 and 1.0 mM drove appetitive feedings in *H. armigera* caterpillars [Figure 10A; paired-sample *t*-test: 0.1 mM, *t*(135) = −4.403, *P* < 0.0001; 1.0 mM, *t*(97) = −3.415, *P* = 0.001], but 0.1 mM and 1.0 mM gossypol drove aversive feedings in *H. assulta* caterpillars [Figure 10A’; paired-sample *t*-test: 0.1 mM, *t*(99) = 3.268, *P* = 0.001; 1.0 mM, *t*(137) = 2.179, *P* = 0.031]. Tomatine at concentrations of 0.01 and 0.1 mM drove appetitive feedings in *H. armigera* caterpillars [Figure 10B; paired-sample *t*-test: 0.01 mM, *t*(103) = −2.371, *P* = 0.02; 0.1 mM, *t*(114) = −3.324, *P* = 0.001], while 0.1 mM and 1.0 mM tomatine significantly deterred feedings of *H. assulta* caterpillars [Figure 10B’; paired-sample *t*-test: 0.1 mM, *t*(118) = 6.941, *P* < 0.0001; 1.0 mM, *t*(170) = 9.369, *P* < 0.0001].

Nicotine at the concentration of 1.0 mM deterred feedings of *H. armigera* caterpillars [Figure 10C; paired-sample *t*-test: 1.0 mM, *t*(98) = 6.471, *P* < 0.0001] but drove appetitive feedings of *H. assulta* caterpillars at concentrations of 0.1 and 1.0 mM [Figure 10C’; paired-sample *t*-test: 0.1 mM, *t*(101) = −7.569, *P* < 0.0001; 1.0 mM, *t*(110) = −2.916, *P* = 0.004]. Capsaicin at the concentration of 1.0 mM significantly drove aversive feedings of *H. armigera* caterpillars [Figure 10D; one-sample *t*-test: 1.0 mM, *t*(100) = 2.972, *P* = 0.004), while 0.01 and 0.1 mM capsaicin significantly drove appetitive feedings of *H. assulta* caterpillars [Figure 10D’; one-sample *t*-test: 0.01 mM, *t*(90) = −5.727, *P* < 0.0001; 0.1 mM, *t*(100) = −2.412, *P* = 0.018].

## Discussion

### Behavioral and Gustatory Response to the Primary Metabolites

Fructose, glucose, and proline have been widely reported to be phagostimulants for a variety of insect herbivores (Albert et al., 1982; Bernays and Chapman, 2001; Liscia et al., 2004; Jiang et al., 2015; Mang et al., 2016). Our present study also shows that fructose could drive appetitive feedings of caterpillars in both *Helicoverpa* species. Glucose and proline at the given concentrations drove appetitive feedings of *H. assulta* caterpillars but had no significant effects on the generalist species *H. armigera*, suggesting that the generalist is less sensitive to the two compounds. This result is consistent with our previous study that the feeding preference of *H. armigera* caterpillars is more flexible than that of *H. assulta* if caterpillars pre-exposed to different diets (Wang et al., 2017). The neural constraint hypothesis predicts that specialist herbivores always make more accurate decisions than generalists in the process of selection plants (Bernays, 1998). Our data provide further evidence that the specialist had better ability to perceive the sugars or essential nutrients than the generalist. However, our result indicated that the responding patterns of GRNs in galeal sensilla to each primary metabolite were similar between the two species, suggesting that the difference of feeding preferences should not be attributed to the firing rate of peripheral GRNs but might be from differences of the processing information within the central nervous system.

### Behavioral and Gustatory Response to the Secondary Metabolites

Gossypol and tomatine are two major plant secondary metabolites from cotton and tomato, respectively, which are toxic or aversive on herbivorous insects (Vickerman and de Boer, 2002; Mulatu et al., 2006; Arnason and Bernards, 2010; Carriere et al., 2019). Our results also show that the two compounds drove aversive feedings of the specialist *H. assulta*, but caterpillars of the generalist *H. armigera* exhibited appetitive feedings for the two secondary metabolites. Such kind of secondary metabolites drove appetitive feedings of the generalist herbivores; to our knowledge, they have not been reported to date. We postulate that it should be attributed to the extraordinary adaptive capacity of caterpillars of *H. armigera* to the two compounds, for example, the tolerance and detoxifying metabolism (Mao et al., 2007; Zhou et al., 2010; Bretschneider et al., 2016; Krempl et al., 2016), while caterpillars of the specialist *H. assulta* do not feed on cotton and tomato plants in nature (Mitter et al., 1993) and exhibit aversive responses to the two secondary metabolites.

Nicotine (Szentesi and Bernays, 1984; Shields et al., 2008; Hori et al., 2011; Sollai et al., 2015) and capsaicin (Cowles et al., 1989; Hori et al., 2011; Li et al., 2020) have been generally reported as feeding deterrents for herbivorous insects. However, our results demonstrate that the two solanaceous alkaloids elicited appetitive feedings of the specialist *H. assulta*, while they drove aversive feedings of the generalist *H. armigera*. We also postulate that it could be attributed to the specialist *H. assulta* being more adaptive to the two alkaloids than the generalist *H. armigera*. Firstly, it is known that tobacco and hot pepper are two limited host plants of the specialist *H. assulta* (Mitter et al., 1993), while the generalists have to deal with lots of toxic plant metabolites based on the neural-constraint hypothesis (Levins and Macarthur, 1969; Bernays and Wcislo, 1994; Bernays and Funk, 1999). Secondly, the adaptations of specialists to nicotine and tobacco plants have been well reported on caterpillars of the tobacco cutworm *Manduca sexta* (Snyder et al., 1993; Glendinning, 2002; Wink and Theile, 2002; Govind et al., 2010; Kumar et al., 2014). For capsaicin, it has been found that the larval development of *H. assulta* could benefit from the dietary capsaicin compared to the negative effects on *H. armigera* (Ahn et al., 2011; Jia et al., 2012). At the level of metabolism, the capacity of degrading the capsaicinoids in *H. assulta* was overall higher than that in *H. armigera* (Zhu et al., 2020). Then, our data provide further evidence of adaptation of the specialist *H. assulta* to the toxic plant metabolites at the behavioral and chemosensory levels, which is similar to the attractive effects of “token stimuli,” the specific secondary metabolites from host plants, on other investigated specialist herbivores (Renwick and Lopez, 1999; del Campo et al., 2001; Miles et al., 2005; Sollai et al., 2018).

For the response of galeal sensilla to the four secondary metabolites, it also indicates that each of the four secondary metabolites stimulated different responding patterns of GRNs between the two closely related species. Combining the differences of feeding preferences with the taste response of GRNs of the two species, it suggests that the activities of peripheral GRNs to the four alkaloids could contribute to the difference of feeding behaviors between the two *Helicoverpa* species. Therefore, it seems that the neural coding for behavioral decisions of the investigated secondary metabolites in the two *Helicoverpa* species is different from that for behavioral decisions of the primary metabolites. The present results suggest that the two *Helicoverpa* species evaluate the plant primary metabolites differently at the CNS level, while they evaluate the secondary metabolites differently at both peripheral and central levels.

## Conclusion

In conclusion, our present results show that the difference of both behavioral feedings and electrophysiological responses to plant metabolites between the two *Helicoverpa* species could contribute to the difference of diet breadth in the two species. Especially, it indicates that caterpillars of the specialist *H. assulta* preferred more to glucose and proline than the generalist *H. armigera*, suggesting that specialist herbivores are more efficient in finding food sources than generalists. More interestingly, gossypol and tomatine, the two secondary metabolites from host plants of the generalist, could drive appetitive feedings of this insect species, suggesting that generalist insects adapt not only to toxic secondary metabolites at metabolism level but also at the behavioral and chemosensory levels.

We also found that nicotine and capsaicin, the secondary metabolites from two limited host plants of the specialist *H. assulta*, could drive appetitive feedings of this insect herbivore, suggesting that this specialist also has adapted to its host plants at behavioral and gustatory levels. However, it is not clear why the generalist *H. armigera* did not prefer nicotine and capsaicin since tobacco and hot pepper plants are also the host plants of this generalist species. We postulate that it may be related to the host plant shifts, host adaptations, fitness costs, and evolutionary pressures during the evolution between *Helicoverpa* species and their host plants. Regardless, our finding would give a new insight of underscoring the adaptation of generalist insects to its host plant. In addition, in future work, the ecological context of the evolution and the further adaptation mechanisms of *H. armigera* to these compounds should be addressed.

## Data Availability Statement

The original contributions presented in the study are included in the article/supplementary material, further inquiries can be directed to the corresponding author/s.

## Author Contributions

QT, WH, and YM conceived the experiment. LS, WH, and JZ conducted the experiment. QT, LS, and XZ wrote the manuscript. LS, QT, and WH analyzed the data. YM, YD, and QY edited the manuscript. All authors read and approved the final manuscript.

## Conflict of Interest

The authors declare that the research was conducted in the absence of any commercial or financial relationships that could be construed as a potential conflict of interest.
